# Effect of Chitosan Nanoparticle from *Penaeus semisulcatus* Shrimp on *Salmonella typhi* and *Listeria monocytogenes*

**Published:** 2020-02

**Authors:** Sogol ALEBOUYEH, Mehdi ASSMAR, Mirsasan MIRPOUR

**Affiliations:** Department of Microbiology, Faculty of Basic Sciences, Lahijan Branch, Islamic Azad University, Lahijan, Iran

**Keywords:** Shrimp, Chitin, Chitosan nanoparticles, *Listeria monocytogenes*, *Salmonella typhi*

## Abstract

**Background:**

After cellulose, chitin is one of the most important polymers in crustaceans, insects, and fungi. Chitosan is one of the most important derivatives of chitin, which has important characteristics including degradability, non-toxicity, and biocompatibility antimicrobial and antioxidant properties.

**Methods:**

Chitosan was extracted from *Penaeus semisulcatus* shrimp using chemical methods and the degree of its austenitization was determined using a sub-red spectrophotometer and XRD. The nanoparticles were then synthesized using the ionic gelation method and analyzed through SEM. The antimicrobial effects of nanoparticles were also evaluated using antimicrobial tests on *Listeria monocytogenes* and *Salmonella typhi.*

**Results:**

Nanoparticles have antimicrobial activity and can inhibit bacterial growth at different concentrations.

**Conclusion:**

Chitosan nanoparticles have an inhibitory effect on *Listeria monocytogenes,* which is a gram-positive bacterium.

## Introduction

Resistance to human pathogens is a major challenge in the pharmaceutical and medical fields. These antibiotic resistances and the persistent consumption of chemical drugs have resulted in the important phenomenon of resistance in microorganisms (1). With the development of this phenomenon, the effect of drugs is weakened or even neutralized, ultimately causing increased drug consumption and the tendency to use compounds with newer and stronger formulation (2). Another disadvantage of using these drugs is their increased side effects, leading to the reappearance of Multiple Drug Resistance (MDR) and parasites (3, 4). Antibacterial agents are very important in various industries, including disinfection of water, textiles, packaging, construction, medicine, and food (5, 6).

Organic compounds traditionally used for disinfection in many industrial processes have various disadvantages including toxicity to the human body and high sensitivity to high temperatures and pressures (5, 7). For this reason, many studies have begun to increase the use of inorganic disinfectants such as metal oxides (8, 9). These inorganic compounds exhibit strong antibacterial activity at low concentrations. Meanwhile, nanotechnology is one of the fastest growing parts of advanced technology. Products containing nanoparticles can be used in various industrial, medical, personal and military applications (10).

One of the areas of extensive research on the application of mineral nanoparticles is the possibility of using them as a disinfectant for controlling microorganisms. The insolubility of the materials used in food packaging is one of the biggest problems of today's society. As a possible solution, the use of biopolymers to eliminate this material is significant (11). Chitosan is very important in this regard, which is a water-soluble organic polysaccharide biopolymer produced through deacetylation of Alkine Ketan units. Recent evidence has revealed that the *Aspergillus Niger* fungus is a source of Chitosan (12).

This biopolymer is a polycationic compound with a special structure and properties, containing more than 5,000 units of glucosamine amine. Chitosan is insoluble in many solvents, but it is soluble in dilute organic solvents such as acetic acid, formic acid, succinic acid, lactic acid, and malic acid (13). This low-cost, non-toxic polymer enjoys decomposability and is environmentally friendly (14).

Due to having active groups, amino is useful as a chelating agent. The degree of acetylation and PH determine the chitosan load (15). At lower PHs, most amino groups protonated in the C-2 position from glucosamine units react with the anionic surface of the lipopolysaccharide of the gram-negative bacteria, as well as with the anionic peptidoglycan of the gram-positive bacteria (16). Therefore, it has antimicrobial activity (17). Reducing PH and increasing the degree of deacetylation potentiate this activity. Therefore, it has been used to control bacterial contamination of food. The aim of this study was to investigate the antimicrobial activity of chitosan nanoparticles against two food-borne pathogenic bacteria to be used in antimicrobial and biodegradable food packaging.

## Materials and Methods

First, 50 ml of the Muller Hinton Agar culture medium was made (1.9 g of powdered Muller Hinton Agar medium, mixed with 50 ml distilled water in Erlon and placed on a flame to completely dissolve the powder). The medium was then sterilized in an autoclave (exposed to a temperature of 121 °C for 15 min, until all bacteria were killed). Next, the culture medium was removed from the autoclave and exposed to room temperature to reach 40 °C (so that it could withstand the temperature of the Arlene). Finally, the medium was melted into the plate to cool completely.

### Utilized microorganisms

Microbial strains including *Listeria monocytogenes PTCC 116* and *Salmonella typhi PTCC 1609* were prepared from lymphocyte collection center of Iran. Each vial was broken under sterile conditions, and for strain activation, the contents of each vial in torn tubes each containing 10 ml of Tryptic Soy Broth medium was transferred to activate the bacteria, where bacteria were incubated for 24 h at 37 °C. After the mentioned time, each of the activated bacteria was transferred to a specific culture medium (McFarland, Muller Hinton broth) and incubated again for 24 h at 37 °C. Finally, to determine the purity of the bacteria and to confirm the strains, diagnostic and confirmatory tests were conducted.

### Chitosan extraction steps

First, 2 kg of green shrimp (*Penaeus semisulcatus*) was purchased containing its head, shell, and tail. For the transfer and to prevent corruption, it was received as frozen. The waste was defrosted and the head and tail sections were separated. Next, the remaining crust was washed for 24 h in brine for further disinfection. Then, it was placed on a clean cloth in order for its water to completely dry. The shells were then placed in an oven at 45 °C for 48 h until they were completely dry finding a crisp and fragile form. The dried crust was crushed into powder by an electric mill and kept inside the chamber at room temperature.

### Extracting Chitin

Initially, 10 g of powdered shrimp was stirred in 150 ml of HCl 4% for 30 min in a shaker incubator at 25 °C. The solution was then centrifuged for 2000 min to purify for 15 min. Next, the powder was washed frequently using distilled water to neutralize and then was evaluated by a pH meter. The neutralized powder was placed inside the oven at 45 °C for 24 h to dry completely. Finally, the resulting powder was weighed using a balance.

### Extracting Chitosan

At this stage, extraction of quinine powder with a weight/volume ratio of 1:20 was performed using NaOH 45% solution at 90 °C for 30 min. Finally, using distilled water, the powder was neutralized and tested by a pH meter it turned out. The powder was then placed in an oven at 45 °C for 24 h to dry completely and then the powder was weighed. The resulting number was 4.5.

### Determining the microbial susceptibility

This test investigated the minimum concentration of chemicals inhibiting the growth of bacteria of food contamination index. For this purpose, different concentrations of nanoparticles were used to determine the minimum inhibitory concentration and bactericidal concentration. These concentrations were 0.10, 0.20, 0.40, 0.80, 1.20, 1.60, 2, 4, 8, 16, and 20 mg/ml. They were poured in microplate of the medium of Muller Hinton broth and microorganisms were placed in a shaker-incubator at 37 °C for 24 h. For each of the bacteria, 20.1 diluted McFarland was inoculated by as much as 20 μl, 100 μl of each dilution of Muller Hinton broth medium and incubated for 17 h at 35 °C for 17 h. In each series of diluted nanoparticle forms, one positive control (including bacteria + medium), control (including nanoparticles + media) and a blank of microplate reader was considered in each period. In order to determine the minimum amount of MBC excretion, a pre- and post-MIC concentration was cultured and incubated at 37 °C for 24 h in a plate containing nutrient agar.

### X-ray diffraction pattern analysis

The sample was sent to the specialized laboratory for analysis of the XRD device. The sample was put into a very small powder and exposed to a bombardment of X-rays, which was the result of a diffraction pattern or diffractogram. Each crystal sample has a unique diffraction pattern identified by comparing it with standard diffraction patterns.

### Production of chitosan nanoparticles

Initially, 20 mg of chitosan was dissolved in 40 mg of acetic acid 1%, where the solution was completely clear and colorless. The pH of the solution was then changed to 5.5 using NaOH solution (5% mol). Next, sodium tripolyphosphate 25% solution (25 mg sodium tripolyphosphate powder in 100 ml distilled water) was prepared. Then, 2 ml of sodium tripolyphosphate solution was added dropwise to 12 ml of chitosan solution at room temperature and stirred rigorously for a period of 1 h to form the nanoparticles completely. Then, centrifugation was done for about 45 min at 5000 rpm to form the nanoparticles. The nanoparticles were dried and the nanoparticles prepared as powder. Afterward, 8.00 gr of powder was weighed in 1 ml distilled water. The nanoparticles were then filtered using a syringe filter and used for microbial tests.

## Results

### SEM

**Fig. 1:**
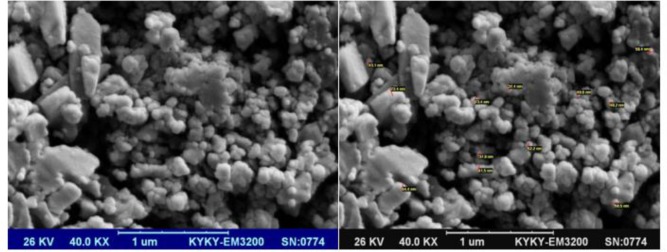
SEM image of synthesized chitosan nanoparticle

The sample surface was composed of dense masses with a size of approximately 22–80 nm distributed uniformly. At first glance, there was a difference between the result obtained by the FTIR, the size of the grains, and the SEM images (Fig. 1). However, the images of the electron microscopy are contingent upon the level of agglomeration.

### FTIR

**Fig. 2:**
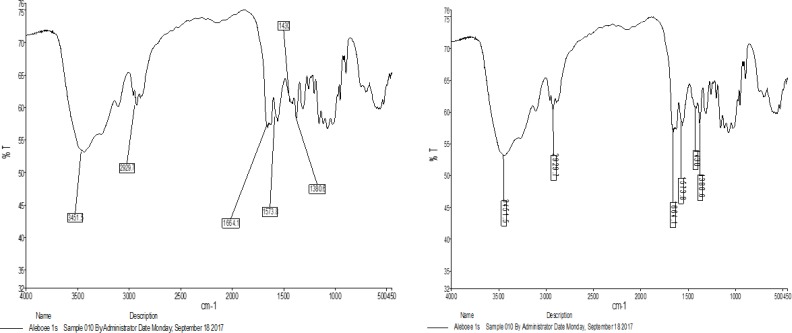
Analysis of Chitosan Nanoparticles Using FTIR

The FTIR histogram confirmed the spectrum of chitosan nanoparticles (Fig. 2), which contains free amine groups, hydroxyl and ether groups. The absorption of an area of about 13400 cm is related to the water contained in 2916 2886 and the material.

The absorption in the area of 1089 is relevant. The courier in the 1380 cm^−1^ area refers to the CH and the ether groups. Courses in the region of C-O 1423 cm^−1^ groups are tensile groups and 1300 cm^−1^ to alcohol and amine groups in the 1700 area.

The presence of the Azomethine group, obtained from the reaction between chitosan and glutenaldehyde, can easily be obtained from the corresponding peak of 1680 cm^−1^ to 1620 cm^−1^, which is indeed a C=N range.

### Determining MIC and MBC

The measured values of MIC and MBC for each bacterium are presented in Tables 1 and 2. The MIC after 24 h for *Listeria monocytogenes* was 0.8 mg/ml. Moreover, the MBC was 1.2 mg/ml, at which the bacterial removal rate was 85.81%. Notably, at the concentration of 16 mg/ml, the removal rate was 100%.

**Table 1:** MIC and MBC Listeria monocytogenes

***Delete percentage***	***Opacity after nano effect***	***Opacity before the nano effect***	***Concentration of mg / ml***
0	1.472	1.472	B1
37.84	0.195	1.472	0.10
67.66	0.476	1.472	0.20
77.58	0.33	1.472	0.40
84.92	0.222	1.472	0.80
85.80	0.209	1.472	1.20
87.02	0.191	1.472	1.60
91.03	0.132	1.472	2
92.39	0.112	1.472	4
99.25	0.011	1.472	8
100	0	1.472	16
100	0	1.472	20

**Table 2:** Effect of Nanoparticle Concentration on *Salmonella typhi* Isolated from Food

***Delete percentage***	***Opacity after nano effect***	***Opacity before the nano effect***	***Concentration of mg / ml***
0	0.675	0.675	B
52.30	0.322	0.675	0.10
61.34	0.261	0.675	0.20
78.52	0.145	0.675	0.40
83.41	0.112	0.675	0.80
88	0.081	0.675	1.20
94.22	0.039	0.675	1.60
97.63	0.016	0.675	2
98.82	0.008	0.675	4
99.85	0.001	0.675	8
100	0	0.675	16
100	0	0.675	20

As reported in Table 2, the lowest inhibitory concentration of 2mg/ml and the lowest concentration that kills bacteria is 4mg / ml. At this concentration, the bactericidal rate was 98.81%, which increased to 100 at a concentration of 16. Finally, Diameter of inhibitory zone of chitosan nanoparticles was compared with antibiotics effective on *Salmonella typhi* and *Listeria monocytogenes* as shown in Table 3.

**Table 3:** The diameter of the growth hole of antibiotics affecting target bacteria according to the CLSI method (mm)

***Antibiotics***	***Salmonella typhi***	***Listeria monocytogenes***
Imipenem	14	23
Piperacillin	16	31
Cefotaxime	22	27
Erythromycin	0	17
Sulfamethoxazole	9	29
Cefazolin	0	21
Cefotetan	0	16
Amoxicillin	0	25
Chloramphenicol	12	20
Gentamicin	12	32
Ticarcillin	18	28
Rifampicin	14	23
Ceftazidime	8	18

### X-ray diffraction pattern

**Fig. 3:**
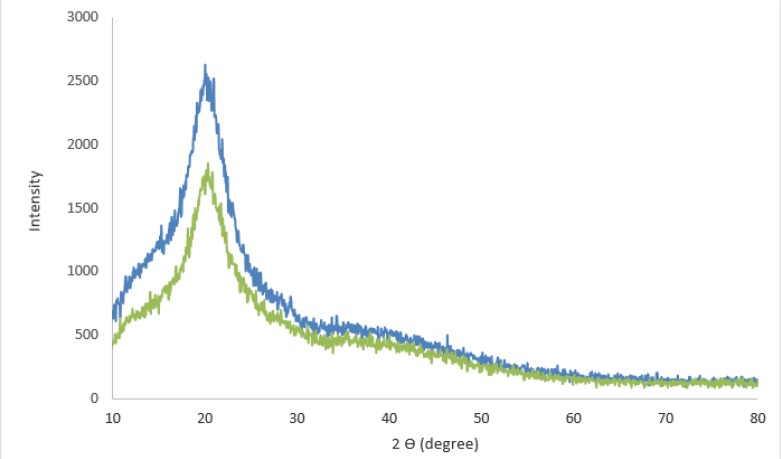
The average sensitivity coefficient for each of the bacteria and nanoparticles investigated in this study

The x-ray diffraction pattern showed that the nanoparticles synthesized by the strains are of chitin. The results were matched by X-ray diffraction spectroscopy. Figure 3 shows an elemental map of the x-ray diffraction pattern of the distribution of chitosan. In these images, the chitosan presence locations (green line) are fully matched to standard samples (blue line).

## Discussion

The study of bacterial insufficiency caused by acidic and water-soluble chitosan concentrations showed that the greatest inhibitory effect was observed in acid-soluble chitosan 10%, while the minimum effect occurred on water-soluble chitosan. Chitosan showed the highest growth inhibitory effect on *Listeria monocytogenes*, while it had the least effect on *Salmonella typhi*. Compared to common antibiotics, in some cases, acid-soluble chitosan, to some extent as with common antibiotics, prevented bacterial growth. In other words, the bacteria resistant to common antibiotics were sensitive to chitosan (18).

Substances used in the food industry should be degradable and have no detrimental effects on the food (19). This is the reason why chitosan has been chosen as a natural biopolymer in this field (20). Lefeng et al. synthesized chitosan nanoparticles of 80 nm in size and reported that the concentration of 1 μg/ml was considered as the concentration of MIC and MBC for pathogenic bacteria (17).

In the present study, this concentration was found to be 20 μg/ml for the pathogenic bacteria. One of the reasons for the difference at this concentration can be the difference in the size of the resulting nanoparticles (21). The size of nanoparticles is very influential in their antimicrobial effect, and the smaller it is, the greater the antimicrobial effect will be. In addition, the bacterial species is also influential in sensitivity to nanoparticles. The reason for the difference in the size of chitosan nanoparticles can also be attributed to the chitosan type utilized (22).

Chitosan, with a wide range of antimicrobial activity, exhibits a different inhibitory effect on fungi, gram-positive and gram-negative bacteria (23). Antibacterial activity is a complex stage that differs between gram-positive and gram-negative bacteria due to their cell surface specification (24). Chitosan has been reported to have a stronger bactericidal effect on gram-positive bacteria than on gram-negative bacteria (25). This might be due to the external membrane dam of gram-negative bacteria (26). In the present study, Listeria monocytogenes, the inhibitoriest bacteria, was a gram-positive bacterium (24).

The initial distillation rate of chitin was above 89%, which justifies the antimicrobial activity of chitosan. A higher degree of degradation with a positive charge had been successful, especially in inhibiting the growth of S. aureus. The antibacterial activity of chitosan against bacteria grows with increasing the degree of deacetylation (27). In this study, water-soluble chitosan showed good activity against *Salmonella typhi* and Listeria monocytogenes, indicating the efficacy of the solubilization method. At pH=7, chitosan is not active in terms of antibacterial properties, since at this pH it is not soluble and its amino groups do not have a positive charge (28). Other studies also have indicated the anti-bacterial activity of water-soluble chitosans against gram-positive and gram-negative bacteria.

Iran is a country with great access to marine resources (29). The creation of shrimp waste conversion industries and the production of products such as chitin and chitosan can be an important step towards employment, especially in the southern regions of the country (30).

## Conclusion

The antimicrobial effects of chitosan produced showed that this material could exhibit inhibitory properties against both *Salmonella typhi* and Listeria monocytogenes bacteria. In this regard, the studies on the effect of this substance on the bacteria mentioned showed that chitosan consumption significantly reduced the number of bacteria over time when compared to the control.

## Ethical considerations

Ethical issues (Including plagiarism, informed consent, misconduct, data fabrication and/or falsification, double publication and/or submission, redundancy, etc.) have been completely observed by the authors.
